# Effect of dietary humic substances on rumen fermentation, blood parameters, immune function, milk composition, and mammary health in periparturient dairy cows

**DOI:** 10.5455/javar.2026.m1056

**Published:** 2026-06-22

**Authors:** Šimon Halás, František Zigo, Andrej Récky, Zuzana Lacková, Zuzana Farkašová, Juliana Arvaiová, Petra Timkovičová-Lacková, Dagmar Mudroňová, Jana Záhumenská, Ewa Pecka-Kielb, Aleš Pavlík, Mária Schilling-Tóth Boglárka

**Affiliations:** 1 Department of Animal Nutrition and Husbandry, University of Veterinary Medicine and Pharmacy in Košice, Komenského 73, 041 81 Košice, Slovakia; 2 Department of Microbiology and Immunology, University of Veterinary Medicine and Pharmacy in Košice, Komenského 73, 04181, Slovak Republic; 3 Department of Hygiene, Technology, and Health Food Safety, University of Veterinary Medicine and Pharmacy in Košice, Komenského 73, 04181, Košice, Slovakia; 4 Department of Animal Physiology and Biostructure, Wroclaw University of Environmental and Life Sciences, Norwida 31, 50-375 Wroclaw, Poland; 5 Department of Animal Morphology, Physiology, and Genetics, Mendel University in Brno, Zemědělská 1665/1, Brno, 613 00, Czechia; 6 Department of Physiology and Biochemistry, University of Veterinary Medicine Budapest, H-1078 Budapest, István utca 2, Hungary

**Keywords:** Cows, supplementation, start of lactation, ammonia nitrogen, phagocyte activity, SCC

## Abstract

**Objectives:** The present trial aimed to evaluate the impact of humic substances (HS) on rumen fermentation, blood and immunological parameters, milk yield and composition, and mastitis incidence in dairy cows during the first 50 days of lactation.

**Materials and Methods:** Sixty healthy pregnant Slovak spotted cows in the 2 months preceding calving were allocated to two groups (30 cows per group). The control group (C-group) received total mixed ration (TMR) without HS, while the experimental group (HS-group) received HS at a dose of 100 gm/day per cow for 60 days antepartum (d.a.p.) and 8 days postpartum (d.p.p.).

**Results:** HS supplementation increased ruminal pH and the acetate-to-propionate ratio (C2:C3; 3.46% vs. 2.56%), while ammonia concentrations decreased on the 8^th^ d.p.p. At the same time, animals treated with HS showed reduced serum and milk urea levels. Furthermore, milk protein and non-fat dry matter content increased, while somatic cell count (SCC) decreased. This was reflected in fewer udder quarters infected with mastitis pathogens in the HS group on 8^th^ d.p.p. The positive effect of HS supplementation was associated with a favorable modulation of the ruminal fermentation pattern and reduced irritation by pathogenic bacteria, leading to decreased blood leukocyte concentrations and reduced phagocytic capacity and engulfing ability on 8^th^ d.p.p. in the HS group.

**Conclusions:** As demonstrated in our study, HS can be efficiently used as a promising organic additive in the TMR for both dry and fresh cows as part of a comprehensive herd health program.

## 1. Introduction

The peripartum period in cows is a critical transitional phase marked by intense physiological, metabolic, and hormonal changes associated with calving and lactation. During this time, cows often experience reduced dry matter intake (DMI), negative energy balance (NEB), and immunosuppression. Due to these changes, cows are more susceptible to metabolic disorders such as acidosis and ketosis, as well as to other diseases such as metritis and mastitis [1]. Nutrition plays a vital role during this period, including feeding a balanced diet that supports optimal metabolism and the body’s natural defenses. Additionally, nutritional needs, such as energy and protein, are significantly higher in the final third of gestation as pregnancy progresses, underscoring the cost pregnancy imposes on the cow. The peripartum approach requires increased attention to the animal and maintaining the cow’s health to reduce the negative impacts of this period [2]. Improving health can be achieved by enhancing immunological status, which also supports mammary gland health and milk quality. Growing recognition of how immunity affects dairy cow health and milk production benefits the cow, fetus, and milk consumers [3].

For this reason, experts have focused on optimizing nutrient utilization and fermentation processes while reducing reliance on growth promoters, hormones, and antibiotics in veterinary medicine [4, 5, 6].

Recently, many studies have employed various feed additives (e.g., essential oils, tannins or algal extracts, and fats) to influence rumen fermentation, improve microbial activity, and reduce enteric methane emissions [5, 6, 7, 8, 9]. Among these organic feed additives, clay-derived humic substances (HS) have the potential to enhance animal health, nutrient utilization, rumen fermentation, and performance. Humic substances are natural organic compounds formed through humification, a process in which microorganisms decompose organic matter into materials resistant to further decay. They consist of a complex mixture of compounds, including aromatic rings, carboxyl, and phenolic groups, and are found in large quantities in soil, peat, coal, and certain water bodies [10, 11].

In animal production they are used as natural immunomodulators and detoxifiers. In the digestive tract, they act as an absorbent for heavy metals, pathogenic bacteria, and fungi and create a protective film on the intestinal mucosa that blocks the absorption of toxins and free radicals while supporting the immune system, reducing inflammation, and supplying important minerals [12, 13].

The bioavailability of active ingredients depends primarily on the form and processing of HS; therefore, there are many products available on the market that are based on natural ingredients (grinding) [14] or chemical extraction with strong alkaline agents [15].

As concerns increase about using organic alternatives as feed additives to boost immunity [12], modulate rumen fermentation, and improve nutrient utilization efficiency, HS has become an important option [15]. Previous studies have shown that adding HS positively impacts gut health, stabilizes intestinal flora, and improves nutrient utilization from feed, which in turn influences milk yield and milk efficiency, while also enhancing the nutritional quality of raw milk from dairy cows and goats [7, 13, 16, 17].

However, the use of HS as an organic additive in dairy cows’ diets during pregnancy and the transition period has not been well documented. Knowledge about the effects of HS administration in dairy cows during the peripartum period on rumen content, immunology, and milk composition is limited. Therefore, we hypothesized that supplementing HS during the dry period can improve rumen fermentation efficiency (reducing NH_3_-N), enhance systemic and cellular immune status, and improve milk quality (reducing milk urea; MU, and somatic cell count; SCC), thereby reducing the incidence of mastitis in early and peak lactation.

Thus, the objective of this study was to evaluate whether HS supplementation in dairy cows during the dry period and the first 8 d.p.p. has a positive nutraceutical effect on feed nutrient utilization, immunity via phagocyte activity, biochemical and hematological parameters, milk quality, and the occurrence of mastitis in the first 50 d.p.p.

## 2. Materials and Methods

### 2.1. Ethical approval

For this study, the clinical examination of the cows and sample collection were approved by the Ethics Committee at the University of Veterinary Medicine and Pharmacy in Košice, no. EKVP 2024–2, in accordance with EU legislation 2010/63/EU, article 1:5 (practices not likely to cause pain, suffering, distress, or lasting harm equal to or greater than that caused by the insertion of a needle, following good veterinary practice).

### 2.2. Monitored dairy herd and experimental design

The study was conducted on a dairy farm in the Prešov district of eastern Slovakia from February to May 2024. The herd consisted of 300 Slovak Spotted dairy cows housed in two modern buildings with high-volume ventilation and automated manure removal systems. In the stable, the cows were divided into two sections with free group housing, each equipped with a feeding table. Each section was further divided into four separate subsections: dry cows—60 days after parturition (d.a.p.); pre-fresh cows—21 d.a.p.; fresh cows—calved up to 21 d.p.p.; and lactating cows from 22 to 100 d.p.p. The mean annual milk yield per cow was 7,830 kg. All animals were provided *ad libitum* access to a total mixed ration (TMR) [18]. The basal TMR had a defined ingredient composition (Table 1) and was formulated to meet the nutritional requirements of each stage of pregnancy or lactation [19] (Table 1).

**Table 1.** Formula and nutrient composition of total mixed ration (TMR) for cows.

Components	Dry cows		Pre-fresh cows		Fresh cows		Lactating cows	
kg	C	HS	C	HS	C	HS	C	HS
Corn silage	14.0	14.0	7.0	7.0	14.0	14.0	23.0	23.0
CCM	0.9	0.9	0	0	0.6	0.6	0.9	0.9
Alfalfa silage	12.0	12.0	10.0	10.0	2.5	2.5	4.0	4.0
Grass silage	6.0	6.0	0	0	2.0	2.0	3.6	3.6
Grass hay	1.0	1.0	1.0	1.0	0	0	0	0
Feed straw	2.0	2.0	2.0	2.0	0.4	0.4	0.6	0.6
Concentrate^1^	0.1	0	3.5	3.4	6.0	5.9	10.0	10.0
Brewer’s grain	0	0	0	0	5.0	5.0	8.0	8.0
Humic substances^2^	0	0.1	0	0.1	0	0.1	0	0
Energy supplements	0	0	0.7	0.7	0.6	0.6	1.0	1.0
Composition by analysis								
DM (gm/kg)	394.5	401.0	449.5	453.3	438.3	442.0	434.9	437.2
CP (gm/kg DM)	89.8	87.3	128.0	125.0	154.2	152.8	158.1	156.7
Fat (gm/kg DM)	33.3	32.7	30.6	29.5	59.0	57.5	43.7	44.5
NDF (gm/kg DM)	507.8	504.2	428.2	425.1	312.0	319.7	350.9	348.3
ADF (gm/kg DM)	345.7	347.3	266.6	262.8	187.4	183.8	187.4	188.6
Starch (gm/kg DM)	156.5	154.4	176.1	175.3	360.9	356.2	273.9	272.8
NFC (gm/kg DM)	302.0	301.0	333.0	335.0	397.0	389.6	374.0	373.0
Ash (gm/kg DM)	67.4	68.8	74.6	75.5	78.3	79.0	73.6	74.1
Calcium (gm/kg)	28.1	30.5	31.2	33.7	57.1	61.2	62.15	61.7
Phosphorus (gm/kg)	14.6	13.2	20.5	19.4	28.1	27.2	30.4	29.2
Sodium (gm/kg)	5.1	4.6	8.0	7.4	20.1	18.6	23.2	22.5
Magnesium (gm/kg)	8.3	9.0	12.2	13.0	18.7	20.0	18.0	18.6
Zinc (mg/kg)	420.5	422.2	534.0	537.1	741.3	744.1	809.0	812.0

Note: Concretate^1^ – concentrate mixture ingredients: rapeseed meal, soybean meal, wheat, barley, oat, and corn; Humic substances^2^ – HS supplemented group with addition of HUMAC^®^ Natur AFM., SK at a dose of 100 gm/cow per day for 60 d.a.p. and 8 d.p.p.; C – control group; CCM = corn cob mix; DM – dry matter; CP – crude protein; NDF – neutral detergent fibre; ADF – acid detergent fibre; NFC – nonfibrous carbohydrate; Humic substances^1^ – characteristics of preparations with humic substances.

Slovak spotted cows in the final stage of pregnancy were used as research animals during a four-month experimental phase consisting of selection of dairy cows, supplementation with HS, and evaluation of fermentation, blood, immunology, milk parameters, and occurrence of mastitis. Out of 140 inseminated and sonographically confirmed dairy cows from the entire herd, only gravid dairy cows in the production age between 2 and 4 lactations were selected. The selected 60 dairy cows met the criteria for creating two homogeneous experimental groups, consisting of clinically healthy animals from the last two months before calving, with SCC below 200,000 on the last two milk utility controls and a negative California mastitis test (CMT). Also, cows were not treated with antibiotics during lactation or drying therapy. The drying protocol consists of the application of teat sealant as a keratin plug (Teatseal, Zoetis, NZ) with the immersion of the teats in a protective solution (T-Hexx Dry External Teat Dip, Zoetis, NZ) without administration of intramammary antibiotics.

Selected 60 cows were divided into two groups: control (C) and experimental (HS) and placed in free group housing of 30 animals per group under the same microclimatic conditions. The animals were fed a TMR twice daily, at 06:00 am and 3:00 pm, with feed refusals maintained at approximately 5% of the daily dry matter intake (DMI). Water was provided *ad libitum*. The HS group was supplemented with HS (see Table 2; HUMAC^®^ Natur AFM, SK) at a dose of 100 gm/cow per day for 60 d.a.p., including the first 8 d.p.p. The daily dose of humates for 30 dairy cows was 3 kg, which was prepared by mixing with concentrate. This was then mixed with other feed components (Table 1) in a vertical feeder wagon, and a complete TMR was dosed onto the feeding table for the dairy cows.

**Table 2.** Characteristics of preparation with humic substances.

	HUMAC^®^ Natur AFM^1^		
parameter		mineral content	mg/kg
particle size	up to 100 μm	Ca	42.3
pH	5.8	Na	7.12
moisture	max. 21%	K	903
humic acids	min. 65% in DM	Br	54.7
fulvic acid	min. 5% in DM	Mg	5.11
crude fiber	24.3 gm/kg	Fe	19.05
ash insoluble in HCl	3.69%	Cu	15
		Zn	37
		Mn	142
		Co	1.24
		Se	1.67
		Mo	2.7

Note: HUMAC^®^ Natur AFM (HUMAC sro., SK)^1^ – The production is based on the processing of humic acids from Leonardite (highly concentrated mineral source of organic substances) through mechanical transformation without adding chemical substances or stabilizers.

After 8^th^ d.p.p., HA supplementation was stopped, and the cows from the maternity ward were moved to the production group with the rest of the milking cows and milked together into the bulk tank cistern. All cows from the HS and C groups were monitored on the 8^th^ day postpartum (fresh cows) and 50^th^ d.p.p. (lactating cows).

### 2.3. Sampling of blood, ruminal content, and TMR

On the 8^th^ and 50^th^ d.p.p., blood, ruminal content, and milk samples were collected from all dairy cows in each group. Blood samples were taken from the jugular vein to analyze the hemato-biochemical profiles. Blood collected in EDTA and heparinized vials was used for hematological investigations and phagocyte activity testing, while serum was separated from blood without anticoagulants for biochemical profiling. At the same time after calving, rumen fluid was collected. Rumen fluid samples for analysis of fermentative and synthesizing capacity were collected 4–6 h after the morning feeding using an oro-ruminal probe and preserved with thymol.

All blood and rumen fluid samples were transported in coolers with ice packs to the Laboratory of the University of Veterinary Medicine and Pharmacy in Košice (UVMP), Slovakia. Blood samples were centrifuged at 3000 rpm for 10 min. Serum was separated and frozen at –20°C to collect the minimum number of samples needed for the biochemical sets of the Cobas chemistry analyzer (Roche Diagnostics, Basel, Switzerland). Hematology analyses and phagocyte activity were performed on whole blood within 2 h of collection. TMR samples were collected into plastic bags according to Maskaľova et al. [20], reflecting changes in the stage of gravidity or lactation, specifically from dry cows, fed until the 60^th^ day of drying; pre-fresh cows, fed 21 days before planned calving; fresh cows, fed 21 days postpartum; and lactating cows, fed 100 days postpartum.

### 2.4. Udder examination and milk sample collection

All dairy cows from both groups underwent clinical examination, udder palpation, and milk sampling to detect mastitis on the 8^th^ and 50^th^ d.p.p. The modified California Mastitis Test (CMT) is a cow-side diagnostic tool used to detect mastitis by assessing SCC in milk. It involves collecting milk from each quarter into a four-well paddle, mixing it with an equal volume of detergent reagent, and checking for gel formation or discoloration, which indicates DNA release from somatic cells [21]. Afterwards, milk samples from each quarter were collected to identify udder bacterial pathogens as described in [22]. Each positive mastitis case was assigned a grade according to the National Mastitis Council guidelines [23], with severity categorized as subclinical or clinical. For analysis of the physicochemical composition, milk sampling followed the principles of STN EN ISO 707 [24]. All samples were transported in coolers with ice packs to the UVMP Laboratory in Košice, Slovakia.

The production capacity of dairy cows, but especially the economic profit, plays a very important role for cattle owners. In the case of our study, we determined the yields from milk sales using the formula:

\[
{\mathrm{Current \;selling \;price \;of \;milk \;per \;liter }} \times {\mathrm{ number \;of \;liters \;per \;animal }} \times {\mathrm{ number \;of \;days}}
\]

Costs associated with the administration of humic acids were 100 gm/animal/day and were subsequently subtracted from the achieved yields using the following formula:

\[
{\mathrm{animals}}\; \times \;{\mathrm{number \;of \;days}}
\]

### 2.5. Sample analysis

#### 2.5.1. Analysis of rumen fermentation characteristics

Samples of rumen fluid were strained through 4 layers of gauze, centrifuged for 25 min, and diluted 1:50. Volatile fatty acids (VFAs) such as acetate (C_2_), propionate (C_3_), and butyrate (C_4_) in the rumen content were determined in a two-capillary isotachophoresis analyzer EA 100 (VILLA LABECO, Slovak Republic). Rumen pH was measured with a portable electronic pH meter (JP SE-LECTA, Spain), and ammonia (NH_3_) levels in the samples were determined as Kjeldahl-N using a 2300 Kjeltec Analyzer Unit (Foss Tecator AB, Hoganas, Sweden).

#### 2.5.2. Hematological and biochemical parameters

Hematological parameters were measured in blood samples of the whole blood on the IDEXX ProCyte analyzer (IDEXX GmbH, Germany): the number of erythrocytes (RBC), leukocytes (WBC), and platelets (PLT); hemoglobin concentration (HGB); hematocrit value (HKT); and erythrocyte indices (MCV—mean corpuscular volume, MCH—mean corpuscular hemoglobin, and MCHC—mean corpuscular hemoglobin concentration).

A trio of analytical methods, laser flow cytometry, impedance, and colorimetry, were used to determine a complete blood count (CBC) using a small sample volume (15 µl). These techniques allow rapid, automated, and accurate measurement of WBC, RBC, PLT, and HGB. Serum samples were processed in five-position racks on a Cobas c111 analyzer (Roche Diagnostics, Switzerland). The reaction mixture is automatically aspirated from the measuring cuvettes placed in a 37°C water bath, mixed with the sample and reagent, and then the whole blood sample is tested for total protein (TP), albumin (ALB), blood urea nitrogen (BUN), glucose (GLU), cholesterol (CHOL), bilirubin (BIL), alkaline phosphatase (ALP), aspartate aminotransferase (AST), and gamma-glutamyl transferase (GGT).

#### 2.5.3. Phagocyte activity testing

The percentage of active phagocytes and their particle-engulfing capacity were evaluated using the commercial IngoFlowEx Kit (Exbio Praha a.s., Czech Republic). The assay was performed with freshly collected heparinized blood according to the manufacturer’s instructions. Sample analysis was conducted on a BD FACSCanto flow cytometer (Becton Dickinson Biosciences, Qume Drive, San Jose, CA) equipped with BD FACSDiva software, which uses scatter properties (FSC/SSC) to identify cells, red fluorescence (*FL-2*) to exclude bacterial aggregates based on DNA content, and green fluorescence (*FL-1*) to quantify the percentage of active phagocytes and their ability to engulf particles (mean fluorescence intensity). Granulocytes and monocytes were identified based on their size (forward scatter, FSC) and granularity (side scatter, SSC). Bacterial aggregates, which have lower DNA content than leukocytes, were excluded using a red-fluorescence (*FL-2*) histogram to ensure that only phagocytic cells were analyzed. The percentage of active phagocytes and their engulfing capacity (mean fluorescence intensity) were determined using a green-fluorescence (*FL-1*) histogram, typically tracking labeled (e.g., FITC-labeled) bacteria.

#### 2.5.4. Phenotyping of lymphocyte subpopulations

Flow cytometric analysis of the selected lymphocyte subpopulations in the peripheral blood was performed by direct immunostaining as follows: 25 μl of heparinized blood was incubated for 20 min in the dark at laboratory temperature with a mixture of conjugated monoclonal anti-bovine antibodies (2 μl CD4-FITC, 1 μl CD8-RPE, and 1 μl CD21-AF 647). For analysis, the following monoclonal antibodies were used: mouse anti-bovine CD4 conjugated with FITC, clone CC8, IgG2a (Thermo Fisher Scientific); mouse anti-bovine CD8 conjugated with R-phycoerythrin, clone CC63, IgG2a (Thermo Fisher Scientific); and mouse anti-bovine CD21 conjugated with Alexa Fluor™ 647, clone LT21, IgG1 (Thermo Fisher Scientific). Standard flow cytometry protocols typically involve washing antibody-labeled cells twice with 1 ml of staining buffer (like PBS) at 250 *× g* for 5 min, followed by final resuspension in 100 µl of buffer to remove unbound antibodies and ensure optimal cell concentration for analysis. Lymphocyte proportions in clinical, immunological, and research studies are commonly reported as percentages of the total white blood cell (WBC) count, with the mean and standard deviation used to indicate population averages and variability. Normal lymphocyte levels typically range from 20% to 40% of the total WBC count.

#### 2.5.5. Analysis of milk composition and SCC

To test the basic components of milk from the monitored cows, such as fat, protein, lactose, and non-fat solids (SNF) content, by FTIR spectroscopy using a MilkoScan FT 6000 (Foss Analytical A/S, Hillerød, Denmark) according to Zahumenská et al. [25]. Milk urea (MU) content was measured on a CHEMSPEC apparatus (Bentley Instruments Inc.) according to Pecka-Kiełb et al. [26]. The somatic cell count was determined in fresh milk using a LactoScan SCC analyzer (Milko-tronic Ltd., Nova Zagora, Bulgaria), a direct-fluorescence instrument equipped with a low-magnification microscope, fast auto-focus, and cell-counting software, enabling rapid and accurate SCC determination in accordance with the relevant international standards—STN EN ISO 13366-2:2010 [27]. All measurements were performed twice for each sample.

#### 2.5.6. Bacteriological examination of milk samples

To isolate bacteria such as *Staphylococcus* sp., *Streptococcus* sp., *Enterococcus* sp., *E. coli*, and *Bacillus* sp., samples were initially cultured on Columbia agar with 5% sheep blood (Oxoid Ltd., Basingstoke, Hants, UK) and incubated at 37°C for 24 h. For precise pathogen identification, colonies were subcultured onto selective media, including Edwards medium, Staphylococcal Medium No. 110, and MacConkey agar (Oxoid Ltd., Basingstoke, Hants, UK), and incubated for an additional 24 h. Following standard protocols for catalase testing, hemolysis assessment, pigment production, coagulase testing, and Gram staining, species were identified according to the methods published by Holko et al. [22].

Biochemical tests, including ENTEROtest 24, STAPHYtest 24, and STREPTOtest 24 (Erba Lachema, Brno, Czech Republic), were used for bacterial identification with TNW v7.0 ProAuto software (Erba Lachema, Brno, Czech Republic). All presumptive staphylococci and streptococci identified by biochemical testing were subsequently confirmed using a matrix-assisted laser desorption/ionization time-of-flight (MALDI-TOF) Biotyper (Bruker Daltonics, Leipzig, Germany). Mass spectrometry measurements were performed on bacterial extracts prepared according to Ozbey et al. [28].

#### 2.5.7. Analysis TMR

Samples were analyzed for dry matter (DM), crude protein (CP), fat, acid and neutral detergent fiber (ADF, NDF), starch, and ash, using conventional methods according to Commission Regulation [29]. The DM content of the samples was determined by oven-drying at 105 ± 2°C. The Kjeltec type 2300 (Foss Tecator AB, Höganas, Sweden) was used to determine the crude protein content according to the Kjeldahl method using a Kjeltec type 2300 (Foss Tecator AB, Höganas, Sweden).

The Soxhlet method was used to determine total fat (as ether extract). Fat was extracted with petroleum ether in a 2-unit extractor (Det Gras J.P., Selecta S.A., Barcelona, Spain). ADF and NDF were extracted by defatting the well-dried samples and separating the residue in a fiber extractor (Dosi-Fibre extractor, J.P. Selecta S.A., Barcelona, Spain). Ash content was determined by burning the samples at 550°C in an AAF 1100 electric muffle furnace (Carbolite Gero, Derbyshire, United Kingdom); starch content was determined polarimetrically using an automatic polarimeter (AP-300, Atago, Japan), and some minerals were determined by the method of atomic absorption spectrophotometry according to Illek et al. [30]. Non-fiber carbohydrates (NFC) were calculated according to the standard NRC [19].

### 2.6. Data analysis

The data collected from both groups were initially processed using Microsoft EXCEL^®^ and Statistica™ software. Prior to analysis, the normality of distribution was assessed using the Shapiro-Wilk test to ensure compliance with parametric assumptions. Statistical evaluation was performed using the General Linear Model procedure in the Statistical Analysis System software. A two-way ANOVA for repeated measures was employed to evaluate the main effects of treatment (HS-supplemented vs. control group), time point (8^th^ vs. 50^th^ d.p.p.), and their mutual interaction. This model was chosen to account for the dependency between repeated measurements taken from the same animals. Dependent variables included all parameters of rumen fermentation, hematology, blood biochemistry, cellular immunity, and milk composition. Differences between specific group means were further analyzed using Tukey’s post-hoc test. The prevalence of mastitis and distribution of pathogens were analyzed using the Chi-square test. Statistical significance was set at *p* < 0.05. All data are presented as means ± standard deviations.

## 3. Results

### 3.1. Effects of HS on ruminal fermentation constituents

The statistical module using a two-way analysis of variance (ANOVA) for independent groups confirmed that HS had a demonstrable positive effect on the stabilization of the rumen environment (Table 3). In the postpartum period, on 8^th^ d.p.p., we confirmed a significantly lower pH (6.37, *p* < 0.05) in the rumen content of dairy cows in the control group (C) compared to those in the group whose TMR was supplemented with humic acids (HS). Conversely, the ammonia nitrogen (NH_3_-N) concentrations in the rumen content of cows in the C group were higher (15.77 vs. 16.15, *p* < 0.05) on the 8^th^ d.p.p. and 50^th^ d.p.p. compared to the HS group of cows (12.37 vs. 12.56, *p* < 0.05). The total volatile fatty acids (VFA) were numerically higher in the C group but not statistically significant (*p* > 0.05); however, the proportions of acetic acid (C2; 64.88 vs. 65.53, *p* < 0.05) and propionic acid (C3; 27.44 vs. 28.02, *p* < 0.05) were significantly higher throughout the observed period. At the peak lactation period (50^th^ d.p.p.), similar results were confirmed as in the postpartum period, when higher NH_3_-N content (16.15, *p* < 0.05) with a higher proportion of C_2_ (64.88 vs. 65.53, *p* < 0.05) and C_3_ (27.44 vs. 28.02, *p* < 0.05) was recorded in the C group. The C_2_:C_3_ ratio was lower (2.56 vs. 2.50, *p* < 0.05) in the C group in both periods compared to the HS group (3.46 vs. 3.10, *p* < 0.05) (Table 3).

**Table 3.** Rumen content.

Parameter	group	Fresh cows (8^th^ d.p.p.) x ± SD	Lactating cows (50^th^ d.p.p.) x ± SD
pH	HS	6.83 ± 0.1^a^	6.80 ± 0.2
	C	6.37 ± 0.4^b^	6.65 ± 0.15
NH_3_-N mg/100 ml	HS	12.37 ± 1.7^a^	12.56 ± 1.4^a^
	C	15.77 ± 1.1^b^	16.15 ± 2.1^b^
Σ VFA mmol/l	HS	86.89 ± 7.5	85.23 ± 16.5
	C	104.69 ± 21.3	105.44 ± 19.0
C_2_ mmol/l	HS	60.22 ± 6.4^a^	52.68 ± 10.1^a^
	C	64.88 ± 9.6^b^	65.53 ± 9.7^b^
C_3_ mmol/l	HS	17.47 ± 1.7^a^	23.40 ± 6.2^a^
	C	27.44 ± 10.9^b^	28.02 ± 9.5^b^
C_4_ mmol/l	HS	9.21 ± 1.9	9.14 ± 2.3
	C	12.37 ± 3.2	11.89 ± 3.1
C_2_:C_3_	HS	3.46 ± 0.4^a^	3.10 ± 0.4^a^
	C	2.56 ± 0.6^b^	2.50 ± 0.6^b^

Note: p.p. – postpartum; HS – supplemented group with addition of HUMAC^®^ Natur AFM., SK at a dose of 100 gm/pc per day for 60 d.a.p. and 8 d.p.p.; NH_3_-N – ammonia nitrogen; Σ VFA – sum of volatile fatty acids; C_2_ – acetic acid; C_3_ – propionic acid; C_4_ – butyric acid; C_2_:C_3_ – ratio of acetic acid and propionic acid; ^a,b^, means within a column with different superscripts differ significantly (*p* < 0.05).

### 3.2. Effect of HS on some hematological and serum biochemical parameters

Total mathematical-statistical characteristics of the hematological values studied are given in Table 4. The determined blood parameters, including red blood cell count, hemoglobin, and hematocrit, were within physiological values and were not affected by the dietary HS treatment. On the other hand, in the HS supplemented group, their lower values of white blood cells (WBC) were recorded on the 8^th^ d.p.p. compared to the control group (6.4 vs. 7.7, *p* < 0.05). Although WBC values were lower in the HS-supplemented group, they remained within the physiological range, excluding immunosuppression in the cows during the study period. Evaluating the erythrocyte indices, increased values of MCH and MCHC were observed in both groups on the 8^th^ d.p.p. compared to the physiological range. On the same day, PLT values were lower (244.3 vs. 237.7, *p* < 0.05) in both groups (HS vs. C) than the physiological range (Table 4).

**Table 4.** Hematology.

Parameter/Unit/Physiological range	group	Fresh cows (8^th^ d.p.p.)	Lactating cows (50^th^ d.p.p.)
		x ± SD	x ± SD
WBC [10^3^/mm^3^] < 5.0;10.0 >	HS	6.4 ± 0.78^a^	7.5 ± 0.81
	C	7.7 ± 1.05^b^	8.4 ± 1.16
RBC [10^6^/mm^3^] < 5.0;10.0 >	HS	6.1 ± 0.52	6.0 ± 0.45
	C	5.7 ± 035	5.8 ± 0.41
HGB [gm/l] <9.0;14.0>	HS	11.9 ± 0.65	11.4 ± 0.61
	C	11.1 ± 0.47	11.3 ± 0.51
HCT [%] < 28.0;38.0 >	HS	29.1 ± 2.3	30.4 ± 3.8
	C	31.5 ± 2.7	29.0 ± 2.1
PLT [10^3^/mm^3^] < 300;800 >	HS	244.3 ± 49.3	360.7 ± 36.1
	C	237.7 ± 38.6	339.5 ± 40.2
MCV [fl] < 46.0;65.0 >	HS	50.9 ± 2.18	51.5 ± 3.49
	C	49.7 ± 2.95	50.2 ± 2.86
MCH [pg] < 11.0;17.0 >	HS	20.3 ± 2.3	16.8 ± 2.45
	C	19.6 ± 2.31	16.2 ± 3.12
MCHC [gm/l] < 31.0;34.0 >	HS	38.8 ± 1.3	34.0 ± 2.6
	C	37.5 ± 2.2	33.7 ± 2.9

Note: p.p. – postpartum; HS – supplemented group with addition of HUMAC^®^ Natur AFM., SK at a dose of 100 gm/pc per day for 60 d.a.p. and 8 d.p.p.; C – control group; X – mean value; SD – standard deviation; WBC – white blood cells; RBC – red blood cells; HGB – haemoglobin; HCT – haematocrit; PLT – platelets; MCV – mean corpuscular volume; MCH – mean corpuscular haemoglobin; MCHC – mean corpuscular haemoglobin concentration; ^a,b,^ means within a column with different superscripts differ significantly (*p* < 0.05).

The effects of HS on the biochemical blood profile are presented in Table 5. The concentrations of TP, ALB, GLU, CHOL, and BIL in blood serum were within physiological ranges in both groups throughout the post-calving monitoring period, with no significant differences between the HS and C groups. Moreover, humate supplementation decreased blood urea nitrogen (BUN) concentration compared to the control on the 8^th^ d.p.p. (4.1 vs. 4.94, *p* < 0.05). The comparison of the HS vs. C group showed BUN values above the physiological maximum on the 50^th^ d.p.p. (4.61 vs. 5.05), which may be related to the ration’s high crude protein content. Enzymatic activity of AST was slightly elevated above physiological values in both groups on the 8^th^ d.p.p. and 50^th^ d.p.p. For the other two enzymes (GGT and ALP), their values were within the physiological range and were not affected by the dietary HS treatment (Table 5).

**Table 5.** Biochemistry.

Parameter/Unit/Physiological range	group	Fresh cows (8^th^ d.p.p.)	Lactating cows (50^th^ d.p.p.)
		x ± SD	x ± SD
TP [gm/l] < 60;80 >	HS	72.4 ± 1.5	72.6 ± 2.1
	C	73.0 ± 2.3	73.5 ± 1.6
ALB [gm/l] < 30;40 >	HS	33.6 ± 0.77	33.2 ± 1.05
	C	34.8 ± 0.61	34.5 ± 0.75
BUN [mmol/l] < 3.3;5.0 >	HS	4.10 ± 0.32^a^	4.61 ± 0.35
	C	4.94 ± 0.41^b^	5.05 ± 0.48
CHOL [mg/dl] < 77.3;150.0 >	HS	72.5 ± 3.4	80.1 ± 4.6
	C	74.2 ± 4.1	83.4 ± 3.7
GLU [mmol/l] < 2.2;3.3 >	HS	3.09 ± 0.21	2.89 ± 0.15
	C	3.12 ± 0.13	2.68 ± 0.09
BIL [µmol/l] < 8.5	HS	4.13 ± 0.32	4.01 ± 0.31
	C	3.73 ± 0.38	3.66 ± 0.45
AST [µkat/l] <1.334	HS	1.49 ± 0.11	1.35 ± 0.10
	C	1.52 ± 0.17	1.47 ± 0.24
GGT [µkat/l] < 0.834	HS	0.38 ± 0.09	0.4 ± 0.05
	C	0.42 ± 0.07	0.52 ± 0.06
ALP [µkat/l] < 5.0	HS	0.81 ± 0.1	0.63 ± 0.1
	C	0.76 ± 0.08	0.57 ± 0.05

Note: p.p. – postpartum; HS – supplemented group with addition of HUMAC^®^ Natur AFM., SK at a dose of 100 gm/pc per day for 60 d.a.p. and 8 d.p.p.; C – control group; X – mean value; SD – standard deviation; TP – total protein; ALB – albumin; BUN – blood urea nitrogen; CHOL – cholesterol; BIL – bilirubin; AST – aspartate aminotransferase; GGT – gamma glutamyl transferase; ALP – alkaline phosphatase; T4 – thyroxin; ^a,b,^ means within a column with different superscripts differ significantly (*p* < 0.05).

### 3.3. Cellular immune response

**Figure 1.**
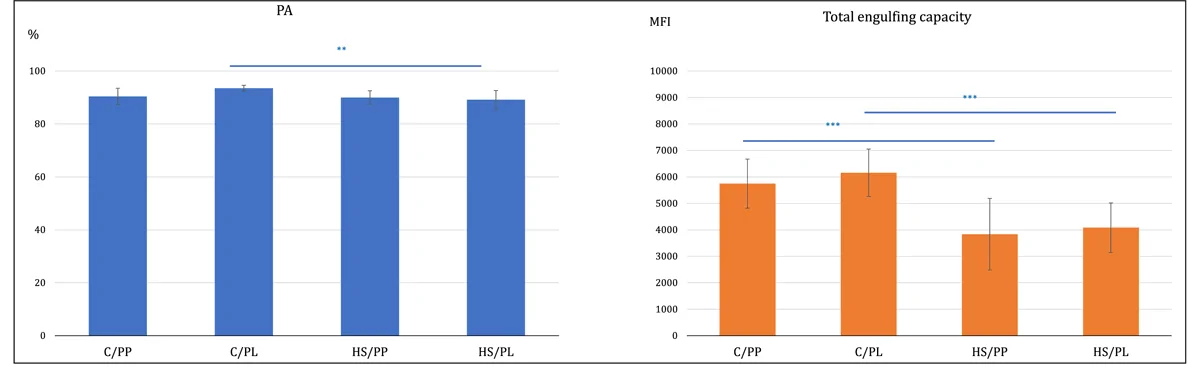
Effect of humic substances on the phagocyte activity (PA; %) and total engulfing capacity expressed as mean fluorescence intensity (MFI) in cows’ blood. Note: PP – 8^th^ d.p.p.; PL – peak of lactation (50^th^ d.p.p.); HS – supplemented group with addition of HUMAC^®^ Natur AFM., SK at a dose of 100 gm/pc per day for 60 d.a.p. and 8 d.p.p.; C – control group; Column labelled with asterisk is significantly different from the control (*p < 0.05, **p < 0.01 or ***p < 0.001).

**Figure 2.**
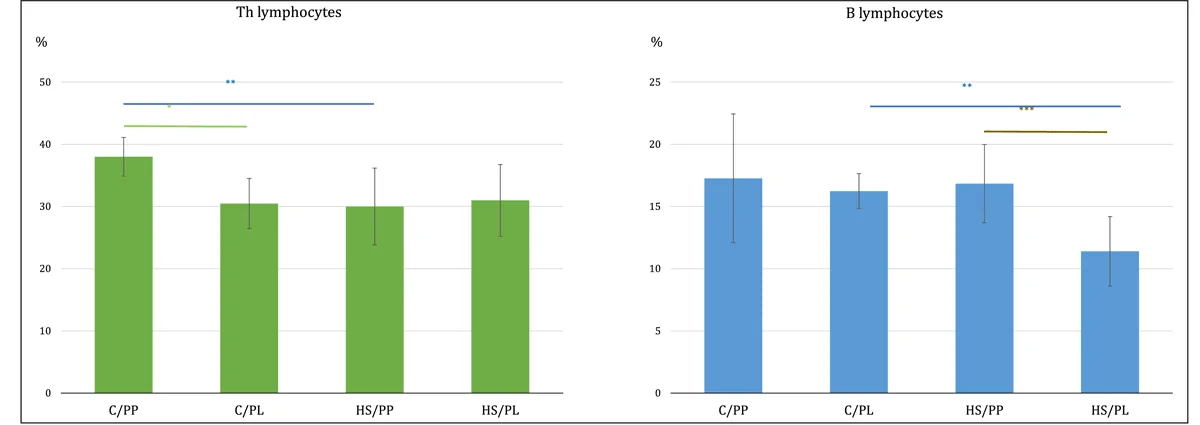
Effect of humic substances on the percentage of selected lymphocyte subpopulations. Note: PP – 8^th^ d.p.p.; PL – peak of lactation (50^th^ d.p.p.); HS – supplemented group with addition of HUMAC^®^ Natur AFM., SK at a dose of 100 gm/pc per day for 60 d.a.p. and 8 d.p.p.; C – control group; Column labelled with asterisk is significantly different from the control (*p < 0.05, **p < 0.01 or ***p < 0.001).

The percentage of phagocyte activity (PA) and the phagocyte engulfing capacity were selected as indicators for monitoring the effect on innate cellular immunity (Figure 1). The addition of HS to cows’ feed has a significant impact on the decrease of PA on the 50^th^ d.p.p. (*p* < 0.05) and the engulfing capacity of the phagocytes on the 8^th^ d.p.p. and 50^th^ d.p.p. (*p* < 0.001), which is associated with a positive effect of stabilizing the ruminal environment and less immunostimulation due to inflammatory changes in the body. The results of Figure 1 also showed a decreasing tendency in PA and the engulfing capacity of phagocytes in both groups on the 8^th^ d.p.p., as at the peak lactation period. The percentages of selected lymphocyte subpopulations are presented in Figure 2. Subpopulation Th lymphocyte (Figure 2) was lower in the supplemented group of dairy cows on the 8^th^ d.p.p. compared to the control group (*p* < 0.01).

**Figure 3.**
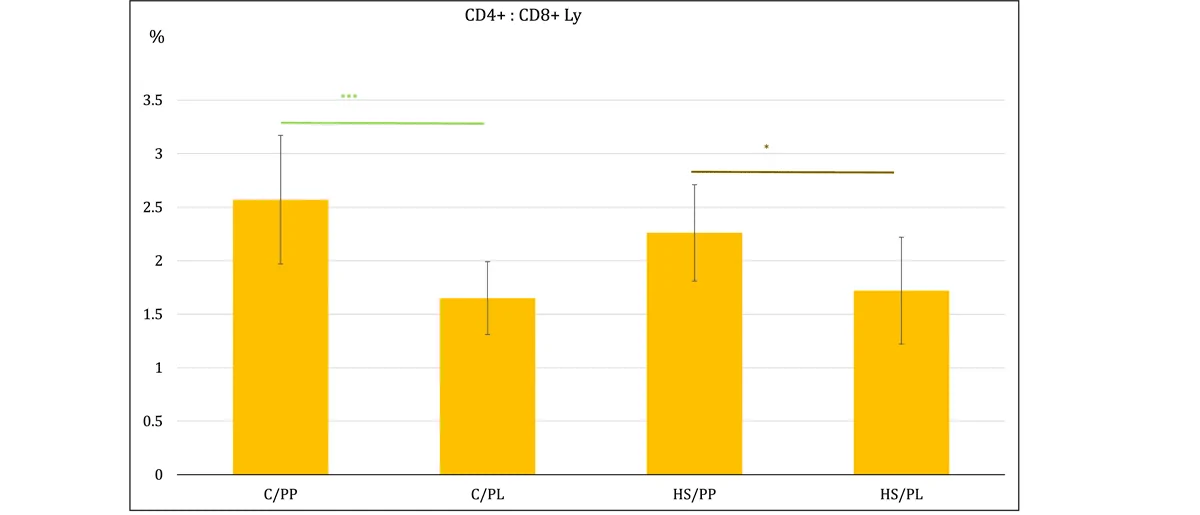
Effect of humic substances on the ratio of CD4:CD8 lymphocytes in the blood of the cows. Note: PP – 8^th^ d.p.p.; PL – peak of lactation (50^th^ d.p.p.); HS – supplemented group with addition of HUMAC^®^ Natur AFM., SK at a dose of 100 gm/pc per day for 60 d.a.p. and 8 d.p.p.; C – control group; Column labelled with asterisk is significantly different from the control (*p < 0.05, **p < 0.01 or ***p < 0.001).

On the other hand, the subpopulation of Th lymphocytes was balanced in the HS group throughout the entire observed period, which is considered a good sign of the organism’s immune adaptation. In the control group, the subpopulation of Th lymphocytes was highest on the 8^th^ d.p.p. (*p* < 0.05). It was found that the addition of HS to cows’ diets significantly decreased the proportion of B lymphocytes on the 50^th^ d.p.p. compared with the control group (*p* < 0.01). Within each group, the subpopulation of B lymphocytes showed a reduced proportion in the HS group on the 8^th^ d.p.p., as on the 50^th^ d.p.p. (*p* < 0.001). Additionally, comparison of the CD4+/CD8+ lymphocyte ratio, as a marker of immune stimulation, showed a significant increase on the 8^th^ d.p.p. in both monitored groups; however, no significant difference between the groups was observed (Figure 3).

Analysis of the interaction (group × duration of administration × time of measurement) using two-way ANOVA indicated that the effect of HS supplementation depends on the duration of administration and on the time of measurement (8^th^ vs. 50^th^ d.p.p.). The interaction was statistically significant (*p* < 0.001) for total engulfing capacity, which was lower throughout the entire measurement period compared with the control group, despite HS being administered only until day 8 p.p. Although PA was higher in the control group on day 50 p.p., analysis of the interaction between duration of administration and the effect of HS did not demonstrate a significant effect. The results of this interaction analysis indicate that HS does not affect phagocytic activity at any stage of lactation.

### 3.4. Effect of HS on milk yield, composition, and occurrence of mastitis

From the 8^th^ d.p.p. up to the 50^th^ d.p.p., the daily milk yield was recorded in Table 6. On the 8th d.p.p. and 50^th^ d.p.p., the difference in the daily milk yield of HS-group cows was > 2 L higher compared to control cows (28.2 and 34.8 vs. 26.4 and 32.9), but it was not statistically significant (*p* > 0.05). Milk composition appears to be affected by humic substance supplementation as well (Table 6). On 8^th^ d.p.p., there was a significant difference in the amount of milk urea (MU, 22.6 vs. 25.7, *p* < 0.05), which was lower in the HS-supplemented group. On the contrary, milk protein (3.7 vs. 3.2, *p* < 0.05) and non-fat dry matter (9.33 vs. 8.86, *p* < 0.05) increased with administration of HS in comparison to the C-group. However, on the 50^th^ d.p.p., there was no significant difference in the milk components.

**Table 6.** Influence of HS administration on milk yield and composition of cows (*n* = 30 per group).

Parameter	group	Fresh cows (8^th^ d.p.p.)	Lactating cows (50^th^ d.p.p.)
		x ± SD	x ± SD
Daily milk yield (kg)	HS	28.2 ± 3.1	34.8 ± 3.8
	C	26.4 ± 2.7	32.9 ± 3.5
MU (mg/100 ml)	HS	22.6 ± 2.8^a^	29.1 ± 4.1
	C	25.7 ± 3.1^b^	28.2 ± 3.2
DM (gm/100 gm)	HS	12.9 ± 0.75	12.45 ± 0.85
	C	12.67 ± 0.82	12.36 ± 0.74
SNF (gm/100 gm)	HS	9.33 ± 0.47^a^	8.65 ± 0.54
	C	8.86 ± 0.32^b^	8.70 ± 0.65
Fat (gm/100 gm)	HS	4.15 ± 0.42	3.84 ± 0.54
	C	4.0 ± 0.35	3.7 ± 0.57
Protein (gm/100 gm)	HS	3.7 ± 0.25^a^	3.5 ± 0.32
	C	3.2 ± 0.28^b^	3.4 ± 0.28
Lactose (gm/100 gm)	HS	4.91 ± 0.29	4.82 ± 0.32
	C	4.84 ± 0.37	4.75 ± 0.30

Note: p.p. – postpartum; HS – supplemented group with addition of HUMAC^®^ Natur AFM., SK at a dose of 100 gm/pc per day for 60 d.a.p. and 8 d.p.p.; C – control group; MU – milk urea; DM – dry matter (total solids); SNF – non-fat dry matter (solids non-fat); *p < 0.05 – significant difference; ^a,b,^ means within a column with different superscripts differ significantly (*p* < 0.05).

The somatic cell count with CMT interpretation is presented in Table 7. The SCCs in the C-group were numerically higher but not statistically significant (295, *p* < 0.05) on the 8^th^ d.p.p. than in the HS-supplemented cows (257, *p* < 0.05). The difference in SCC was not significant on the 50^th^ d.p.p. From a total of 120 tested udder quarters in each group (30 dairy cows), a numerically lower number (8 vs. 14, *p* < 0.05) of positive quarters with a CMT score of 1–3 was recorded in the HS group on the 8^th^ d.p.p. compared to the C group (8 vs. 15, *p* < 0.05). From CMT-positive dairy cows, quarter milk samples were collected and tested in both groups, from which bacterial mammary gland pathogens were identified, namely coagulase-negative staphylococci (*S. warneri, S. xylosus*, and *S. chromogenes*), *S. aureus, Str. fecalis*, and *Aerococcus viridans*. In the 8 d.p.p., a lower number (5 vs. 7, *p* < 0.05) of positive samples cultured for udder bacterial pathogens was observed in the HS-group compared to the C-group; however, their species diversity was not demonstrated (Table 7). The identified udder pathogens in both monitored groups showed the numerically higher occurrence of subclinical mastitis but not statistically significant (*p* < 0.05) on 8^th^ d.p.p. (23.3% in C-group vs. 16.7% in HS-group).

**Table 7.** Influence of HS administration on SCC, CMT evaluation and occurrence of mastitis.

Status	Fresh cows (8^th^ d.p.p.)		Lactating cows (50^th^ d.p.p.)	
	C	HS	C	HS
	x ± SD	x ± SD	x ± SD	x ± SD
^1^SCC × 10^3^	295 ± 31.1^a^	257 ± 25.3^b^	186 ± 23.7	170 ± 19.2
No. of tested cows/quarters	30/120	30/120	30/120	30/120
Negative CMT	105 (87.5%)	112 (93.3%)	110 (91.7%)	113 (94.2%)
*CMT score 1 – 3	15 (12.5%)^a^	8 (6.7%)^b^	11 (9.1%)	7 (5.8%)
*Infected quarters	14 (11.6%)^a^	8 (6.7%)^b^	10 (8.3%)	8 (5.8%)
^2^Positive cows with SM	7 (23.3%)	5 (16.7%)	5 (16.7%)	5 (16.7%)
^3^Positive cows with CM	1 (3.3%)	0	1 (3.3%)	0
No. and % of udder pathogens isolated from infected quarters				
*S. warneri*	4 (28.6%)	2 (25.0%)	2 (20.0%)	2 (25.0%)
*S. aureus*	3 (21.4%)	3 (37.5%)	3 (30.0%)	3 (37.5%)
*S. xylosus*	2 (14.3%)	2 (25.0%)	1 (10.0%)	1 (12.5%)
*S. chromogenes*	1 (7.1%)			1 (12.5%)
*Str. Faecali*s	2 (14.3%)		2 (20.0%)	
*Aerococcus viridans*	1 (7.1%)		1 (10.0%)	1 (12.5%)
*E. coli*	1 (7.1%)		1 (10.0%)	
Mixed infection^3^		1 (12.5%)		

Note: p.p. – postpartum; HS – supplemented group with addition of HUMAC^®^ Natur AFM., SK at a dose of 100 gm/pc per day for 60 d.a.p. and 8 d.p.p.; C – control group; ^1^SCC – average values of somatic cell count per group; SM^2^ – subclinical mastitis; CM^3^ – clinical mastitis; CMT – California Mastitis Test; Mixed infection^3^ – involve a combination of infection with two bacterial pathogens. ^a,b,^ means within a row with different superscripts differ significantly (*p* < 0.05); *Chi-square test with significant difference *p* < 0.05.

The total profit from the production capacity of dairy cows in the HS group, in the case of our study, represented 412.80 EUR for the farmers. We present the specific calculation: the yields are 34.8 L divided by 30 cows, which is 1.16 L per cow × 50 days, which is 58 L per cow × 0.47 EUR/L, which is 27.26 EUR per cow. Costs are 0.27 EUR per animal × 50 days, which is 13.50 EUR. The profit is therefore calculated by the difference: 27.26 minus 13.50 is 13.50 EUR/per cow. Since 30 animals were monitored, this means 13.76 EUR/cow × 30 pc is 412.80 EUR/50 days.

## 4. Discussion

### 4.1. Effect of HS on ruminal fermentation constituents

The rumen plays a very important role in the life of dairy cows, as it functions as a continuous fermenter. To achieve optimal ruminal fermentation parameters and micro-biota symbiosis, the nutrient content in TMR, along with the relative constancy of the rumen environment’s physicochemical properties, is essential. The analysis of the nutrient content in TMR was within the recommended ranges for the different cow groups, meeting the established nutritional requirements. Throughout the monitored period, pH values for both groups ranged from 6.37 to 6.83, which is within the optimal physiological range for rumen fluid pH 5.5–7.5 [31]. Our key finding revealed that dietary supplementation with HS significantly increased ruminal pH and the acetate-to-propionate ratio, while NH_3_-N concentrations decreased throughout the observed period.

Fermentation in the rumen produces ammonia nitrogen, and in the unionized state, ammonia (NH_3_) is readily transferred across the rumen wall and may be excreted in the urine as ammonium salts, used in transamination to form glutamine, or converted to urea in the liver and excreted or recycled back to the rumen. The ease with which ammonia traverses the ruminal wall into the bloodstream also limits the amounts of dietary non-protein nitrogen that can be fed to ruminants. However, in the ionized state (NH_4_^+^), nitrogen can be utilized by bacteria that tend to produce more propionic acid than acetic acid, which would be beneficial to dairy cows [32]. Our results confirmed that HS have an impact on the total amount of VFA and on the individual acids C_2_ and C_3_ during rumen fermentation. Reductions in NH_3_-N concentration in our study indicate good humate absorption capacity and improved efficiency of the proportion of acetic acid and propionic acid. Suteky et al. [33] achieved similar results in his study, which describes the buffering effect of HS, which contributes to the neutralization of VFA and stabilization of pH in ruminants, thereby reducing the risk of subacute ruminal acidosis, especially with high-concentrate TMRs.

Kholif et al. [17] described the effects of HS supplementation on feed utilization and milk production in Friesian cows during early lactation over a 90-day period. Supplementation with HS at 20 or 40 gm per day improved nutrient digestibility, rumen fermentation, and milk production. In addition, a reduction in ruminal protozoa, NH_3_-N content, and butyric acid concentration was also observed. However, a higher humate dose was recommended over a lower dose. In accordance with the previous study and recommended HS dosages [34], we administered high doses of HS (100 gm of HS/cow per day) to the experimental group. This approach yielded the expected nutraceutical effect, as hypothesized, by reducing NH_3_-N concentration and stabilizing the rumen environment. Evaluating ammonia in the ruminal content of cows showed higher values in the C group than in the HS group on the 8^th^ d.p.p. and 50^th^ d.p.p., indicating that HS can excrete ammonia, thereby reducing its content in the rumen and promoting its utilization by microorganisms for the formation of microbial protein. This leads to more efficient conversion and smaller environmental losses, with a negative impact on ecology [35].

On the other hand, low doses of HS or short administration periods may not yield the expected results. In a study by McMurphy et al. [32], it was determined that HS at doses of 5.0 and 10.0 gm/kg of diet do not alter rumen fermentation in Holstein steers fed high-concentrate diets. After 14 and 21 days of HS supplementation, no treatment differences were observed for total VFA concentrations or percentage proportions of acetate, propionate, butyrate, or valerate, although a quadratic response was observed for butyrate. Váradyová et al. [36] reported that HS at higher dietary levels increased the nitrogen content incorporated by microbiota as well as the efficiency of microbial synthesis. Humic substances in the diet were not effective as antiprotozoal agents; however, they may influence microbial growth and energy efficiency. Therefore, HS preparations could be used as growth promoters in ruminant production at levels up to 10 gm/kg of feed dry matter.

Similar effects on the stabilization of the ruminal environment, including reduced ruminal NH_3_-N concentrations, were reported by El-Zaiat et al. [7], who administered HS orally at a dose of 2 gm/day per goat at 14 d.a.p. and 56 d.p.p. Humic substances increased rumen pH and the acetate-to-propionate ratio, while NH_3_-N concentrations and protozoal counts decreased. The ability of HS to alter ruminal fermentability by sequestering NH_3_-N and subsequently releasing it slowly for microbial growth is supported by an approximately 20% reduction in ruminal ammonia concentration. Consequently, reducing protozoal numbers may increase microbial crude protein flow to the small intestine.

A comparison of our results with other studies [7, 13, 16, 17, 35] indicates that humic substances (HS) have a strong potential to influence rumen fermentation parameters; however, these effects can be inconsistent and vary significantly depending on their source environment, the duration of administration, and the actual dosage of HS consumed.

### 4.2. Effect of HS on blood chemistry measurements and cellular immune response

In the present experiment, the hematological and serum parameters indicated that cows in both groups remained within the normal physiological range. Comparing both groups, the control group exhibited elevated WBC (hematological parameter) and blood urea nitrogen (BUN; serum parameter) on the 8^th^ d.p.p. BUN levels are directly linked to ammonia metabolism, which was influenced by HS supplementation. The lower BUN level in cows fed an HS diet can be explained by lower rumen NH_3_-N concentration, as indicated by more efficient crude protein utilization. Something similar was found in Van Soest’s study [37], who supplemented dairy cows with HS during lactation. In cows treated with HS, a lower BUN value was observed, indicating a more efficient use of dietary crude protein for microbial protein synthesis, likely due to HS’s nitrogen-fixing ability.

El-Zaiat et al. [7] observed that supplementation of humates for goats during the dry period and in the first weeks of lactation improved serum total protein, globulin, and glucose concentrations but decreased BUN, which was also confirmed in our study. In HS-treated goats, a lower BUN value indicates more efficient utilization of microbial protein for synthesis, associated with HS’s nitrogen-binding capabilities. This low value of BUN confirmed the observation of Kholif et al. [17] that lower BUN is usually associated with a lower ruminal NH_3_-N flux to the bloodstream. Thus, a reduction in BUN value could be supported by a lower ruminal NH_3_-N concentration.

On the other hand, Kaneko et al. [38] found no change in serum total protein, protein fractions, or the physiological activities of GGT and ALP, suggesting that humate supplementation had no effect on the nutritional status of the cows, muscle protein catabolism, or kidney function. The addition of humic substances to the feed ration of rams did not significantly change key serum biochemical parameters [15]. Their findings suggest that supplementation of HS in rams resulted in no statistically significant changes among biochemical parameters; however, slight numerical trends indicated decreases in serum cholesterol and ALT, while BUN, total protein, and creatinine showed minor increases.

Although our study did not confirm the effect of HS on other biochemical parameters, in the study by Kholif et al. [17], humate supplementation decreased cholesterol concentrations by 10.5 and 12.5% and triglyceride concentrations by 4.9 and 11% in the low-humic and high-humic treatment groups, respectively, suggesting reduced secretion of triglyceride-rich lipoproteins into the lymphatic system. According to Ho et al. [39], humate substances are known to release Fe from ferritin, thus accelerating lipid peroxidation and reducing blood cholesterol and triglyceride concentrations. On the other hand, Degirmencioglu [40, 41] observed reduced cholesterol levels with humic supplementation in lactating goats, without any effect on total protein, glucose, triacylglycerol, or HDL cholesterol levels.

Among other things, the effect of humate addition on ruminal fermentation and blood constituents, various studies indicate that HS can have an immune-stimulating effect [42, 43, 44, 45]. Our study’s primary objective was to track how HS affected several cellular immune indicators in cows during the first 50 d.p.p. The percentage of active phagocytes and their engulfing capacity were selected as indicators for monitoring the effect on innate cellular immunity. From our results, it was found that the addition of HS to the diets of cows during 68 days significantly decreased phagocytic activity on the 50^th^ d.p.p. as well as mean fluorescence intensity on the 8^th^ d.p.p. and 50^th^ d.p.p., which indicates their positive effect associated with the stabilization of the ruminal environment and less immunostimulation due to various pathogenic agents from the environment and inflammatory changes in the organism.

Reduced cellular activity positively correlates with a decreased WBC, which is directly involved in the body’s immune defense. Decreased WBC with HS supplementation probably relates to the increased ability of HS to reduce the absorption and availability of bacterial endotoxins and inhibit the growth of pathogenic bacteria and molds; therefore, the cells responsible for cellular immunity did not have to be activated to the same extent as in the control group of cows without HS addition. Although a reduced content of WBC was noted in the blood of the HS-supplemented group on 8^th^ d.p.p., these values were within physiological limits, which excludes their immunosuppressive state. Our claims are supported by the study Mudroňova et al. [12], which found that the percentage of lymphocyte subpopulations (CD4 lymphocytes) was significantly decreased for broilers fed HS supplementation (0.8%) compared with the control group. They also noted a significant decrease in cytotoxic T lymphocytes, leading to a statistically altered CD4:CD8 ratio, a marker of immune stimulation.

In addition, humic acids prevent excessive water loss through the intestines and create a protective film on the intestinal mucous membranes against infections and toxins, thereby reducing the production of stress hormones and the activation of cellular defense mechanisms in animals, helping them cope with stress [46]. This statement is also supported by the results of our study on selected lymphocyte subpopulations, which monitors the effect of HS on acquired cellular immunity. Results of the present study showed a significant decrease in Th lymphocytes on the 8^th^ d.p.p. and in B lymphocytes on the 50^th^ d.p.p. when HS was supplemented to the TMR of cows. The exact mechanism of action by which HS affects immune cells remains unclear. However, they are most likely to affect cytokine production. Like our findings, Vetvicka et al. [47] reported a significant increase in *IL-5* and *IL-6* production in mice treated intraperitoneally with HS. These cytokines stimulate B lymphocyte differentiation and growth and increase immunoglobulin secretion. The theory explains that the immunomodulatory effect of HS may result from the formation of humate complexes with carbohydrates that bind to the surfaces of T lymphocytes and NK cells, affecting their functions, including cytokine production, which can further affect other cells of the immune system.

According to Mudroňová et al. [12], HS influences the activity of the immune system mainly at the level of the intestinal mucosa, where they function as modulators of humate complexes that scavenge dangerous substances in the body and neutralize free radicals to protect cells from oxidative stress, thereby helping to prevent inadequate immune responses and autoimmune diseases. This action protects the health of both animals and humans by supporting intestinal health, acting as anti-inflammatory and antimicrobial agents, and strengthening overall immunity.

The immunostimulatory effect from HS supplementation that we expect through the formation of humate complexes with carbohydrates in the body can be influenced primarily by the bioavailability of the administered preparation as well as its dose and duration of supplementation. One of the main factors determining the bioavailability of HS-based products is their processing [48]. The production of used HS in our study is based on the processing of humic acids from Leonardite, a highly concentrated mineral source of organic substances. The manufacturing process of our administered product is strictly mechanical, unlike conventional industrial methods that rely on chemical transformation. This approach preserves the complex, high-molecular-weight structure of humic acids, maintaining their 100% natural status without introducing synthetic reagents or alkaline stabilizers. By avoiding chemical processing, the applied HS-based product maintains a high concentration of natural humic acids (min. 65% in dry matter), thereby maximizing their bioavailability [49].

Likewise, the immunostimulatory effect of HS may be influenced by the animals’ age category and the stage of fattening. In most studies, their positive effect was demonstrated in young individuals during growth and fattening, who are generally exposed to greater stressors such as weaning, changes in feed rations, changes in housing, bedding, etc. [12, 15, 42, 43]. In the study by Bujňák et al. [42], the supplementation of HS to the diet of the weaning piglets had no significant effect on phagocytosis. However, the percentage of active phagocytes as well as their engulfing capacity was numerically higher in piglets supplemented with HS than in piglets in the control group.

As shown in most studies, the effects of HS on phagocytosis and specific antibody immunity are not always the same or precisely understood; therefore, further studies will be needed to examine these mechanisms.

### 4.3. Effect of HS on milk yield, composition, and occurrence of mastitis

The efficiency of milk production is limited by the amount of nutrients ingested and their transformation for milk synthesis. A strategic nutritional goal in dairy farming is to maximize the efficiency of metabolic conversion of carbohydrates and proteins into milk. Protein degradation and the synthetic capacity of the rumen microbiota are limited by nutrient intake from the TMR (energy and protein) and by the stability of the rumen environment [48, 49, 50]. However, changes in feed efficiency induced by HS addition may be largely due to differences in milk composition. This was also confirmed in our study, where, on the 8^th^ d.p.p., lower MU levels were measured, along with increased protein and non-fat dry matter in the HS group. The observed decrease in MU in cows fed a diet with HS can be explained by the lower BUN level, which resulted from the lower ruminal NH_3_-N concentration. In the present study, the MU result fits very well with the reductions in BUN and ruminal NH_3_-N observed. In this respect, it has also been shown that MU is a sensitive indicator of the balance between the value and suitability of digestible crude protein and feed energy, and it helps measure protein utilization efficiency. Enhanced milk components, such as protein and non-fat dry matter, in cows treated with HS are currently connected with better use of rumen parameters, which serve as precursors for their formation.

El-Zaiat et al. [7] also state that the MU content is a good indicator for assessing the energy-protein balance in dairy cow nutrition in relation to performance. Except for the reduction of MU, the same author writes, increased milk fat and protein contents are in the HS-supplemented group of lactating goats. Similar results were confirmed in our study when on the 8^th^ d.p.p., a significant difference was recorded in the lower amount of MU and increased non-fat dry matter and protein in comparison to the C-group. Reduced MU levels indicate support for milk protein production by maximizing microbial protein synthesis with NH_3_-N in the rumen serving as the main nitrogen source. The efficiency of TMR nutritional components in N transformation, as reflected in MU content, appears to be a suitable way to assess nitrogen transformation. If the ration is well balanced, the urea level in milk is in the normal range (2.5–5.00 mmol/l). Increased MU concentration can indicate poorer milk quality, accompanied by rumen alkalosis, indigestion, reduced volatile fatty acid production, and bacterial protein [50]. In our experiment, MU content was in the normal range in both groups during all monitored periods.

Potůčková and Kouřimská [51] state that supplementation with HS at a dose of 200 mg/kg in the diet of lactating cows for 3 months increased milk contents of crude protein, pure protein, and casein without any effect on the concentrations of total solids, solids-not-fat, lactose, or fat. Similar positive effects of HS on milk production and milk components are written in studies of Thomassen and Faust [52] and Islam et al. [46]. Changes in some milk variables are specifically related to the cow’s metabolic status, others are associated with udder health, and some reflect both systemic and mammary gland conditions.

Early lactation cows (0–100 days post-calving) are critical for dairy farm profitability, producing approximately 42–45% of total lactation milk yield. This high-production phase triggers substantial metabolic stress, characterized by severe negative energy balance (NEB), rapid loss of body condition score (BCS) due to lipomobilization, and reduced dry matter intake despite high nutrient demands. The disparity between the high energy demand for milk production and relatively low DMI results in a pronounced energy deficit during the first 4–8 weeks postpartum. To compensate for NEB, cows mobilize body fat reserves, which contributes to a marked decline in BCS. Feed intake does not increase as rapidly as milk production, thereby limiting energy supply [53]. Milk yield typically peaks between 50 and 90 days in milk, placing maximum strain on metabolic processes. Especially during this period, there is an increased incidence of mastitis in dairy cows, leading to damage to the glands in the parenchymatous tissue. Damage to glandular tissue leads to an increase in SCC and a decrease in milk production [54]. Therefore, lower SCC indicates healthier udders and better milk quality, as SCC rises with mastitis [55], as confirmed in our study. The SCCs in the HS group were lower on the 8^th^ d.p.p. than in the C-group of cows. In the same period, a lower number of positive quarters based on CMT was recorded in the HS-group, resulting in fewer positive samples cultured for bacterial udder pathogens and fewer cases of mastitis. These results confirm our hypothesis that humic substances (HS) can improve cow health through their bioactive effects by supporting ruminal fermentation and influencing the formation of immune complexes, thereby reducing the incidence of mastitis in the first stage of lactation.

As demonstrated in our study and others, the preventive effect of HS supplementation on SCC in milk and intramammary infections depends on HS concentration, lactation phase, and the duration of administration. The dose we chose, 100 gm of HS/cow/day for 68 days, had a positive nutraceutical and protective effect against udder bacterial pathogens and can be a valuable strategy to improve gut and mammary gland health, potentially lowering mastitis risk and severity as part of a comprehensive herd health program.

Thomassen and Faust [52] found that supplementing dairy cow diets with 3 gm of HA per kg of dry matter during the first two months postpartum reduced SCC in milk by about 50%. This supplementation aids udder health during the critical early lactation period. Likewise, the authors confirmed that processed humic acids have favorable effects on milk production traits for dairy cattle. Although our study recorded a slight but statistically non-significant increase in daily milk production in the HS group (> 2 L higher compared to control cows) during the monitored period, HS supplementation can assist in improving dairy cow performance and economic profit. At a milk price of 0.47 EUR/L, the value gained for the farmer is 47 EUR per cow during the first 100 days of lactation with the addition of HS. After deducting the costs associated with supplementation at a dose of 100 gm of HS/cow/day (amounting to 27 EUR/cow per 100 days), the net profit is 20 EUR per cow, which represents 2,000 EUR for a herd of 100 animals. As our analysis shows, the costs related to long-term HS supplementation (at least 60 days) are sustainable for farmers and are reflected in improved milk quality and reduced SCC, which is one of the main parameters for assessing milk safety and mammary gland health. Furthermore, an additional economic value of HS supplementation is the reduction in mastitis incidence, as demonstrated in our study. In this way, farmers can save significant resources that would otherwise be spent on treating and caring for mastitic animals.

Lashkova and Petrova [56] observed that adding biochemical fulvic acid to the diet of lactating dairy cows significantly improved milk performance and health. Trials demonstrated a 100% cure rate for clinical mastitis and an 80% cure rate for subclinical mastitis, with milk yield increasing by 13.84%–14.94% and feed conversion improving by 13.79%. As a result of the feeding strategy of adding HS to TMR in dairy herds, the author Islam et al. [46] report a decrease in the number of cases of mastitis in dairy cows from an average of three to four cases per day to 4 cases per month.

## 5. Conclusions

Based on the results obtained in this study, it can be concluded that dietary supplementation at a dose of 100 gm/cow per day for 60 days after parturition (d.a.p.) and 8 days post-partum (d.p.p.) improved pH, ruminal fermentation (ratio of acetic to propionic acid, decreased NH_3_-N content), and some milk components (increased milk protein and decreased MU). Reduced concentrations of BUN and MU, together with increased milk protein synthesis and higher non-fat dry matter content, indicate favorable changes in nutrient metabolism, likely related to stabilization of rumen fermentation and reduced ammonia excretion. Also, their positive nutraceutical effect was manifested by a reduction in infected udder quarters and a decrease in SCC on the 8^th^ d.p.p. Given the broad manufacturer recommendations for humate dosages in cows, which range from 20 to 200 gm/cow per day, our study demonstrated that a dose of 100 gm/cow per day has beneficial effects on stabilizing the ruminal environment and on key indicators of ruminal fermentation, milk production, and udder health. Therefore, we recommend that farmers include HS in the TMR for dry cows as part of a comprehensive herd health program.

## Data Availability

The data presented in this study are available on request from the corresponding author. The data are not publicly available due to privacy.

## List of abbreviations

HS, humic substances; TMR, total mixed ration; d.a.p., day antepartum; d.p.p., day postpartum; C2:C3, acetate-to-propionate ratio; SCC, somatic cell count; DMI, dry matter intake; NEB, negative energy balance; IgM, immunoglobulin M; IgG, immunoglobulin G; HA, humic acid; NH_3_-N, ammoniacal nitrogen; MU, milk urea; CMT, California mastitis test; UVMP, University of Veterinary Medicine and Pharmacy in Košice; VFA, Volatile fatty acids; C2, acetate; C3, propionate; C4, butyrate; MH_3_, ammonia; RBC, red blood cells; WBC, leukocytes; PLT, platelets; HGB, hemoglobin concentration; HKT, hematocrit value; MCV, mean corpuscular volume; MCH, mean corpuscular hemoglobin; MCHC, mean corpuscular hemoglobin concentration; CBC, complete blood count; TP, total protein; ALB, albumin; BUN, blood urea nitrogen; GLU, glucose; CHOL, cholesterol; BIL, bilirubin; ALP, alkaline-phosphatase; AST, aspartate-aminotransferase; GGT, gamma-glutamyl-transferase; *FL-2*, red fluorescence; *FL-1*, green fluorescence; SNF, non-fat solids; DM, dry matter; CP, crude protein; ADF, acid detergent fibre; NDF, neutral detergent fibre; PCR, polymerase chain reaction; VFA, total volatile fatty acids; PA, phagocyte capacity; NH_4_^+^, ammonium ion; *IL-5*, interleukin 5; *IL-6*, interleukin 6; CD4, cluster of differentiation 4; CD8, cluster of differentiation 8; BCS, body condition score; CCM, corn cob mix; NFC, non-fibre carbohydrates; PL, peak of lactation; L, liters; gm, grams.
